# A 14-Bit Hybrid Analog-to-Digital Converter for Infrared Focal Plane Array Digital Readout Integrated Circuit

**DOI:** 10.3390/s24113653

**Published:** 2024-06-05

**Authors:** Douming Hu, Libin Yao, Nan Chen, Jiqing Zhang, Shengyou Zhong, Wenbiao Mao, Fang Zhu, Juan Zhang

**Affiliations:** Kunming Institute of Physics, Kunming 650223, China

**Keywords:** hybrid ADC, SAR ADC, SS ADC, infrared FPA, DROIC

## Abstract

This paper presents a 14-bit hybrid column-parallel compact analog-to-digital converter (ADC) for the application of digital infrared focal plane arrays (IRFPAs) with compromised power and speed performance. The proposed hybrid ADC works in two phases: in the first phase, a 7-bit successive approximation register (SAR) ADC performs coarse quantization; in the second phase, a 7-bit single-slope (SS) ADC performs fine quantization to complete the residue voltage conversion. In this work, the number of unit capacitors is reduced to 1/128th of that of a conventional 14-bit SAR ADC, which is beneficial for the application of small pixel-pitch IRFPAs. In this work, a tradeoff segmented thermometer-coded digital-to-analog converter (DAC) is adopted in the first 7-bit coarse quantization process: the lower 3-bit is binary coded, and the upper 4-bit is thermometer coded. A thermometer-coded DAC can improve the linearity of ADC. Capacitor array matching can be incredibly relaxed compared with a binary-weight 14-bit SAR ADC, resulting in a noncalibration feature. Moreover, by sharing DAC and comparator analog circuits between the SAR ADC and the SS ADC, the power consumption and layout area are consequently reduced. The proposed hybrid ADC was fabricated using a 180 nm CMOS process. The measurement results show that the proposed ADC has a differential nonlinearity of −0.61/+0.84 LSB and a sampling rate of 120 kS/s. The developed ADC achieves a temporal noise of 1.7 LSBrms at a temperature of 77 K. In addition, the SNDR is 72.9 dB, and the ENOB is 11.82 bit, respectively. Total power consumption is 71 μW from supply voltages of 3.3 V (analog) and 1.8 V (digital).

## 1. Introduction

In recent years, there has been a trend in infrared imaging technology to reduce the pixel size, increase the resolution, decrease system costs, and enhance detector performance [1,2]. As infrared systems become increasingly compact, maintaining consistent system performance has emerged as a critical objective. One of the primary strategies to achieve this is to decrease the pixel pitch of the infrared detector while simultaneously enhancing performance through technological advancements [3,4,5,6].

With classical analog outputs, high pixel rates lead to increases in both the number of analog video outputs and the overall detector power consumption. Moreover, the analog detector SNR can be degraded by the required external analog acquisition chain operating at ambient temperature. The noise of external ADCs is comparable with the readout integrated circuit (ROIC) output noise, which degrades the overall SNR. Developing on-chip, high-performance, ultra-low-power analog-to-digital converters (ADCs) has thus become a crucial trend to meet system requirements. The ADC of digital readout integrated circuits (DROICs) is critical to the infrared detector performance.

In [7,8,9], small-pixel digital sensors provide advantages in reducing the size, weight, and power (SWaP), improving noise immunity, simplifying interfacing requirements, and lessening susceptibility to electromagnetic interference (EMI). DROIC, which is based on pixel-level ADC, directly accomplishes analog-to-digital conversion in a pixel array [10,11,12,13]. However, this form of pixel-level ADC is only suited for usage in large-sized pixels.

In small-pixel infrared sensors, column-level digital readout circuits are more common due to spatial constraints since the ADC area is limited to a one-dimensional direction.

The single-slope (SS) ADC is the most widely employed in CMOS Image Sensor (CIS) applications because it can be implemented in a narrow column pitch using simple column circuits consisting of a comparator and a counter [14,15]. However, a major drawback of SS ADCs is their relatively slow conversion speed. Each K-bit A/D conversion requires 2^K^ clock periods compared to only K clock periods for successive approximation register (SAR) ADCs and cyclic ADCs. In high-resolution designs, their slow conversion efficiency significantly limits the readout speed of the array. Due to the high dynamic range of infrared images, DROICs typically require high-resolution ADCs above 14 bits. The conversion speed of a conventional pure 14-bit SS ADC is limited by the counting frequency, resulting in high power consumption. SAR ADCs offer a significant advantage in conversion speed; a 14-bit SAR ADC only requires 14 clock periods for conversion. However, SAR ADCs need calibration in high-resolution designs (e.g., greater than 12 bits). Calibration helps save substantial power in the DAC and comparator by correcting their errors, but the calibration overhead in terms of the area, speed, power, and architectural complexity should be minimized [16]. Furthermore, a 14-bit SAR ADC requires 2^14^ unit capacitors, which consume a larger chip area. When the IRFPA pixel pitch is less than 10 μm, designing and laying out a 14-bit column-parallel SAR ADC becomes a considerable challenge, limiting its application in small pixel pitch IRFPAs.

To overcome the limitations of both SS and SAR ADCs, hybrid and two-step ADCs have been developed [17,18,19,20,21,22]. These include multiple-ramp single-slope (MRSS) ADCs [18] and two-step single-slope (TSSS) ADCs [19,20], which aim to balance the advantages of both approaches. For example, an 11-bit two-step ADC [19] combining a 3-bit SS ADC and an 8-bit SAR ADC can ease capacitor array matching requirements without the need for calibration. A 12-bit TSSS ADC [22] with a constant input-common-mode level resistor ramp employs a current-mode R-2R DAC and a feedback R-string DAC to enhance ADC linearity, but it still requires a redundancy of 1.5 bits. Nonetheless, implementing such designs above a 14-bit resolution remains challenging due to circuit complexity and area constraints. The SAR/SS ADC [21] approach reduces the capacitor area and power consumption by sharing analog circuits and minimizing calibration needs. Despite these advancements, achieving high resolution and low power consumption within narrow readout channel pitches remains complex.

Because infrared images have a high dynamic range, the resolution of an ADC in a DROIC is usually larger than 14 bit. High-resolution ADCs require longer conversion times and higher power consumption. Therefore, for the infrared system DROIC, an ADC with low power consumption, a high speed, and a high resolution is urgently needed. To address these issues, power-efficient and area-efficient ADCs for the digital readout integrated circuit (DROIC) are proposed in this work.

The proposed ADC can be implemented in 10 μm small pixel pitch IRFPAs. It is suitable for a column-parallel ADC of a digital readout integrated circuit for IRFPAs. With the sampling frequency of 120 kSps and low power consumption, differential nonlinearity |DNL| < 1, the proposed ADC is attractive for high-frame-rate digital infrared sensors.

The remainder of this article discusses the architecture, operating principles, and implementation details of this proposed ADC, providing measurement results and comparing them with previous works to highlight its advantages and improvements.

## 2. Architecture of the Proposed ADC

### 2.1. Block Diagram

With the combination of the SAR and SS architecture, the suggested hybrid SAR-SS ADC can achieve both the high linearity of the SS ADC and the high sampling rate of the SAR ADC, which makes it appropriate for IRFPA. This integration retains the individual benefits of each type of ADC while mitigating their drawbacks. A coarse SAR ADC and fine SS ADC share a capacitor DAC, ensuring there is no gain error between the coarse and fine quantization stages.

**Figure 1:**
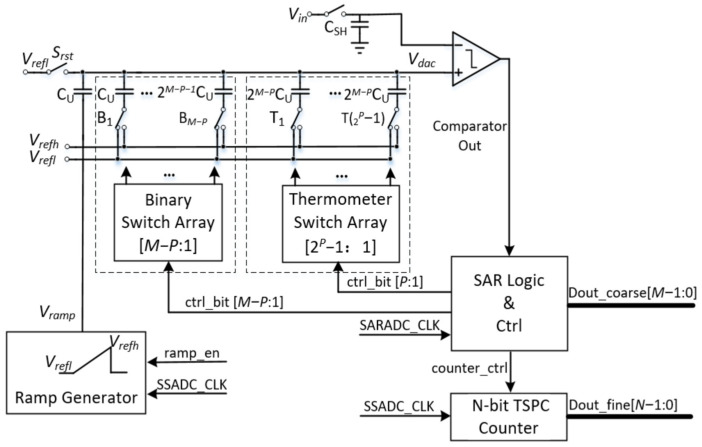
A block diagram of the proposed hybrid ADC and global ramp generator.

Figure 1 shows a block diagram for the proposed two-step column-parallel ADC. The system consists of an N-bit counter, global ramp generator, SAR logic and control circuits, comparator, sample-and-hold circuits, binary switch array, thermometer code switch array, and 2^M^ C_U_ capacitor DAC. The SAR ADC first conducts coarse M-bit quantization, followed by fine N-bit quantization by the SS ADC, resulting in a combined (M + N) bit quantization. V_refl_ and V_refh_ are the reference voltages for the capacitor DAC and ramp generator, respectively. The ramp generating block provides the ramp signal, Vramp. To eliminate linearity errors caused by variations in the ramp signal and the capacitor DAC’s gain error, the ramp signal Vramp is connected to the bottom plate of C_U_ in the capacitor DAC [21]. For every column, the comparator is shared by SAR ADC and SS ADC. Additionally, a global ramp generator is shared by all columns. For each column, the comparator is shared between the SAR ADC and SS ADC. Additionally, a global ramp generator is shared by all columns.

While the binary weighted successive approximation register (SAR) is energy efficient, it is susceptible to errors caused by digital-to-analog converter (DAC) settling and mismatches in DAC capacitors [23]. In contrast, a thermometer code offers several advantages over a binary code, including natural monotonicity, no missing codes, and superior DNL performance. The proposed segmented thermometer-coded capacitor DAC is split into upper P-bit and lower (M-P) bits for use in M-bit coarse SAR quantization, as seen in Figure 1. The upper P-bit section of the capacitor DAC is controlled by a thermometer switch array, while the remaining lower (M-P) bit section maintains a binary switch. This segmented approach allows the higher bits to provide better linearity compared to a purely binary-weighted DAC. By ensuring monotonic features and avoiding glitches caused by voltage peaks, the segmented thermometer-coded DAC can reduce DNL while maintaining the same capacitor area [24]. For instance, an M-bit thermometer-coded decoder would have a 2^M^ − 1 control signal since these require greater space. Given the challenges of implementing a thermometer code decoder in a compact pixel readout channel, this study presents the highest P-bit segment as a feasible solution. In summary, the segmented thermometer-coded DAC improves linearity and reduces DNL, making it a better fit for applications requiring high precision and reliability.

### 2.2. The Operating Principle of the Proposed Hybrid ADC

**Figure 2:**
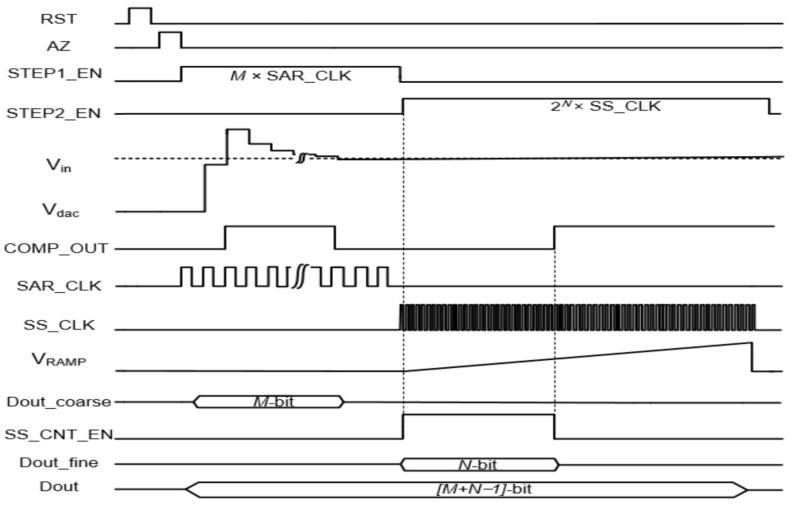
Operational timing diagram of proposed ADC.

Figure 2 depicts a simplified operational timing diagram. In the first phase, coarse quantization is performed by the M-bit SAR ADC, which only requires M clock cycles, making the coarse conversion speed very fast. At each rising edge of the SAR clock, the comparator compares the output of the capacitor DAC V_dac_ to V_in_. Based on the comparator’s output, the SAR logic selects either the V_refl_ or V_refh_ reference voltages to connect to the bottom plate of each capacitor in the DAC. Once the M-bit SAR ADC completes coarse quantization, the residue voltage, V_res_, can be expressed as follows:

(1) $$V_{res}=V_{in}-\sum_{i=0}^{M-1}\left[\frac{\left(V_{refh}-V_{vrefl}\right)}{2^{M-i}}\cdot Dout\_coarse\left[i\right]\right]-V_{vrefl}$$

where Dout_corase[i] represents the i-th bit of the upper M-bit. In the second step, the N-bit SS ADC performs fine quantization to complete the residue voltage conversion. The ramp-generated block produces a ramp signal, V_ramp_, which is connected to the bottom plate of the least significant bit C_U_ in the DAC. During SS ADC quantization, V_ramp_ varies from V_refl_ to V_refh_, consisting of a total of 2^N^ steps with a step size of (V_refh_ − V_refl_)/(2^N^ − 1), and the V_dac_ can be represented as follows:

(2) $$V_{dac}=\sum_{i=0}^{M-1}\left[\frac{\left(V_{refh}-V_{vrefl}\right)}{2^{M-i}}\cdot Dout\_coarse\left[i\right]\right]+V_{vrefl}+\sum_{j=0}^{N-1}\left[\frac{\left(V_{refh}-V_{vrefl}\right)}{2^{\left(M+N-j\right)}}\cdot Dout\_fine\left[j\right]\right]$$

Once the comparator output is triggered, the SS ADC stops counting. As a result, the ultimate outcome of the two-step conversion is obtained, and the (M + N) bit output D can be stated as

(3) $$D=\left\{Dout_{coarse\left[M-1\right]},\ldots ,Dout_{coarse\left[0\right]},Dout_{fine\left[N-1\right]},\ldots ,Dout_{fine\left[0\right]}\right\}$$

The conversion time of the proposed ADC can be briefly expressed as follows:

(4) $${TIME}_{conversion}=M\times SAR\_CLK+2^{N}\times SS\_CLK$$

## 3. The Design of the Proposed Hybrid ADC

### 3.1. The Trade-off between the SAR and SS ADC

**Figure 3:**
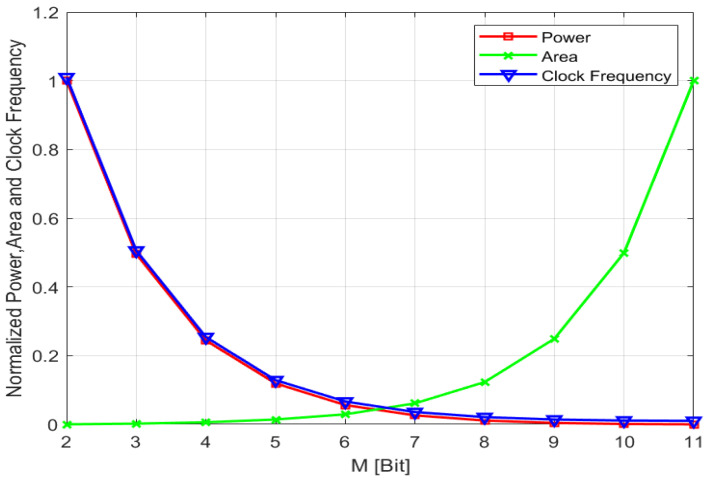
A plot of the power, area, and clock frequency as a function of the M-bit in the first step.

In the proposed 14-bit hybrid ADC, M represents the number of bits in the SAR ADC, while the SS ADC handles the remaining (14-M) bits. Choosing the value of M is crucial. As illustrated in Figure 3, for a 14-bit ADC with a constant sampling rate, increasing M reduces N, which, in turn, lowers the clock frequency and power consumption of the SS ADC. Nonetheless, the area of the capacitor arrays grows exponentially with the M value. When M exceeds 8 bits, the capacitor area increases significantly, complicating the layout.

Since the ADC needs to fit within narrow pitch applications, using an SAR ADC with fewer than 8 bits is more area efficient. As shown in Figure 3, the ADC achieves good power efficiency for both M = 6 and M = 7. Compared to M = 6 and N = 8, where the fine SS ADC takes 2^8^ clock cycles, M = 7 and N = 7 only require 27 clock cycles, giving the M = 7 and N = 7 configurations a speed advantage. Therefore, setting M = 7 and N = 7 is a good compromise, balancing the layout size, power consumption, and conversion speed. This is why this work uses a 7-bit SAR ADC and a 7-bit SS ADC.

The thermometer-coded DAC offers the advantage of having a less strict matching requirement compared to a binary-weighted DAC. A 50% matching of the unit cell is sufficient to achieve a DNL of 0.5 LSB [23]. This allows for good DNL performance within the same area using a thermometer-coded DAC. The image system requirements for INL are generally less stringent compared to those for DNL. For IRFPA applications, ensuring good DNL performance is crucial. However, a significant disadvantage of a thermometer-coded DAC is its increased area. If the number of bits exceeds 4, the thermometer code switch array becomes complex and difficult to implement within a small IRFPA readout channel.

Typically, segmentation is used to strike a balance between the clear advantages of the thermometer-coded architecture and the need to keep the area limited. In this study, a segmented capacitor DAC is introduced. The DAC is divided into two sub-DACs: one for the upper bits and one for the lower bits. For the higher bit, where accuracy is the most crucial, thermometer coding is employed. For instance, a 4-bit binary to thermometer decoder uses 15 control signals for the capacitor switch, which can be implemented in a small channel. However, if a 5-bit de-coder is chosen, the number of control signals increases to 31, making the layout in the ADC channel more challenging. For the 7-bit DAC, using the higher 4 bits with thermometer coding in the 10 μm pitch is a good compromise to balance the circuit area and DNL performance. Therefore, the lower 3 bits use a binary-code DAC, while the upper 4 bits use a thermometer-code DAC. This setup allows the 7-bit SAR ADC to perform coarse quantization in the first phase with only 7 SAR clock cycles. In the second phase, a 7-bit SS ADC conducts fine quantization using 2^7^ SS clock cycles.

The value of the unit capacitor, Cu, is a compromise considering thermal noise, the process capacitance error, parasitic capacitance, and the switching mode of the capacitor array. A higher Cu value improves noise and the matching performance, whereas a lower value is better for power consumption, the chip area, and the settling speed. According to the process parameter of the metal–insulator–metal (MIM) capacitor and Monte Carlo simulation, the matching requirement of the capacitor array can be met with a unit capacitance of 79 fF. Due to spatial limitations and KT/C noise, an MOS capacitor with a capacitance of 2.1 pF has been selected for the sample and hold capacitor.

### 3.2. Comparator

**Figure 4:**
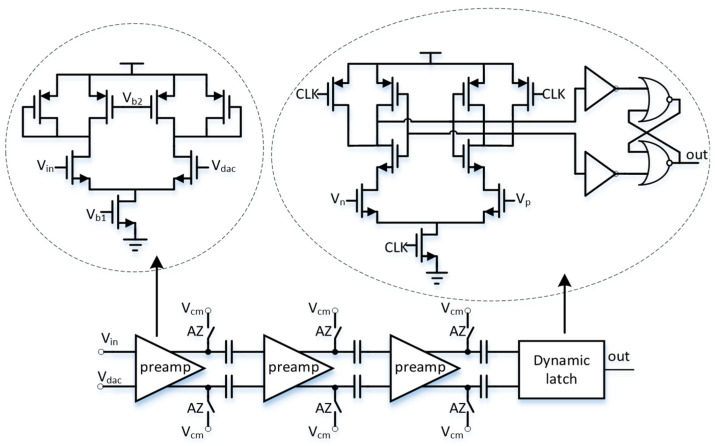
A b lock diagram of the comparator.

A fast and high-precision comparator is essential for high-resolution ADCs. Regenerative comparators with several-stage amplifiers are widely used in pure SAR ADCs [23,25]. Figure 4 shows a simplified diagram of the comparator used in the proposed hybrid ADC. The comparator consists of three-stage amplifiers, a regenerative dynamic latch, and auto-zeroing capacitors. In this study, a three-stage pre-amplifier construction is employed to enhance the open-loop gain for the hybrid ADC application. The pre-amplifier uses a differential amplifier with a current-starved load, providing an easy way to balance bandwidth and gain. Moreover, the comparator’s clock can be regulated using the SAR and SS clocks during the first and second steps, respectively. An auto-zeroing mechanism is also included, which activates at the beginning of the comparison process.

In this design, the comparator is shared by both the SAR ADC and the SS ADC. During the coarse SAR ADC phase, the residual signal decreases as a result of the SAR bit cycling. A regenerative comparator makes the comparison by sensing a minimal static residue voltage in the LSB bit cycle. Sensing and amplifying a smaller residue signal helps extend the decision time needed to determine logical level “1” or “0”. A metastable state occurs if the decision delay is longer than a limited comparison time, which is critical in high-speed SAR ADCs [26]. There is only one comparison required by the SS ADC in the fine SS ADC phase. High-speed, high-precision comparators for SAR logic typically require more current for the amplifiers, leading to significant power consumption. Therefore, the comparator’s power consumption can be efficiently reduced by appropriately relaxing the SAR ADC’s clock frequency. To address this, the period of the SAR clock is set to be four times longer than that of the SS clock. Thus, Equation (4) can be represented by the conversion time as follows:

(5) $${TIME}_{conversion}=7\times 4\times SS\_CLK+2^{7}\times SS\_CLK$$

In this work, the SAR ADC operates at a frequency of 12.5 MHz, while the SS ADC clock’s frequency is 50 MHz. This setup strikes an excellent balance between power consumption and speed, ensuring the linearity of the 7-bit MSB quantization and enhancing the DNL.

The noise from the first-stage pre-amplifier comparator is the primary noise source, so it is crucial to have a relatively high bias current for this stage. The first stage has a quiescent current of 6 μA, while the subsequent stages each have a quiescent current of just 3 μA. To minimize the response delay of the comparator, the 3 dB bandwidth is set to around 100 MHz.

The dynamic comparator has the advantages of low power consumption, a short transmission delay, and a low input offset voltage. It only consumes dynamic power during clock edges, making it highly energy efficient [26]. The dynamic latch performs the timing comparison and generates a digital output based on which input signal becomes high first without consuming any static power. However, the dynamic latch can introduce significant kickback noise, negatively impacting ADC performance [27,28]. To address this, the comparator offsets are cancelled at the start of the conversion process using the auto-zeroing function. This effectively reduces the influence of DC offset voltage in both the latch and the pre-amplifier, improving the overall ADC performance.

### 3.3. Ramp

**Figure 5:**
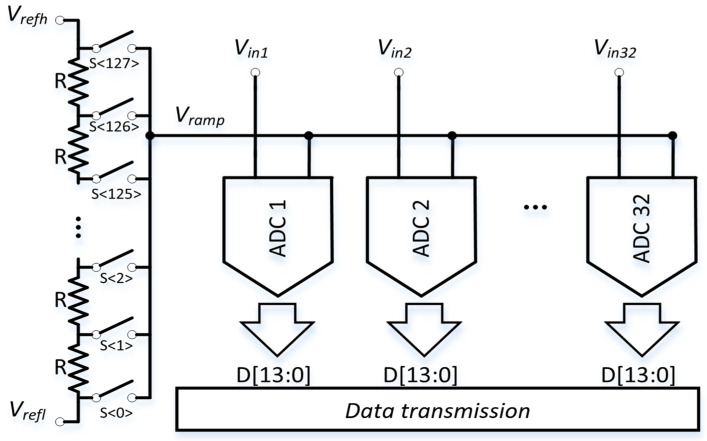
A block diagram of the global ramp generator and proposed column-parallel ADCs in IRFPAs.

As shown in Figure 5, the global ramp generator is shared by all column ADCs in the DROIC IRFPAs. In this multiple-project wafer (MPW), there are 32 ADCs in the array. After the conversion, all of the ADC data are transmitted by the transmission module.

In the second step of the proposed ADC, the ramp is generated by a 7-bit resistor-string DAC (RDAC). For each step of the RDAC, $V_{STEP}=\left(V_{refh}-V_{refl}\right)/127$. The settling time of each RDAC step is given by the following equation:

(6) $$∆V_{RAMP}\left(t\right)=\left(1-e^{-t/\tau }\right)*V_{STEP}$$

where $\tau =R_{driver}\times C_{load}$, and $R_{driver}\approx R1+R_{load}$. R1=1/2(127∗R) in the worst case. $R_{load}$ refers to the metal resistor value of the ramp layout. The total capacitive load of the ramp includes $C_{lumped}$, which represents the parasitic capacitance of the layout, as well as the load capacitance of 32 ADC’s C_U_ array.

(7) $$C_{LOAD}\approx \left(C_{lumped}+32\times C_{U}\right)$$

The RDAC is frequently used due to its simple structure and inherent monotonicity. The theoretical values of the 7-bit RDAC INL is given by $0.5\sqrt{2^{7}-1}\sigma$, where the factor 0.5 comes from the fact that the INL curve was fit to zero at both ends of the curve [24]. Resistors σ is a function of area, $\sigma^{2}\propto 1/area$. The INL requirements for the ADC are more relaxed in the case of image sensors because the pixels contribute more to the nonlinearity of the system. Therefore, the impact of the resistor-string DAC INL on the whole ADC can be neglected.

The ramp generator consists of a resistor string comprising 127-unit resistors, R, each with a resistance of 50 Ω. With the ADC’s sampling rate being 120 kS/s, the SS ADC clock frequency during the fine 7-bit conversion is 50 MHz. This means VRAMP spends 20 ns for each RDAC step. The proposed ADC can quantify the input signal between V_refl_ and V_refh_, which are set to 1.2 V and 3.1 V, respectively. The analog circuitry is powered by 3.3 V, while the digital circuits operate at 1.8 V.

### 3.4. Layout

The number of unit capacitors of the proposed two-step ADC is reduced to 1/128th of that in a conventional 14-bit SAR ADC, making it very area-efficient. The proposed two-step ADC is low in area occupancy, and the layout can be implemented in a pixel pitch of less than 10 μm. This design allows the layout to fit within a pixel pitch of less than 10 μm.

Since the DNL is more sensitive to the capacitor mismatch than the resistive mismatch, the proposed hybrid ADC requires the capacitor DAC to have a careful layout to ensure the accurate conversion of the upper 7 bits. To address this, dummy capacitors are placed on both sides of the 7-bit DAC to minimize capacitance mismatch. Another factor to consider is the effect of oxide thickness gradients over large capacitor areas. As discussed in previous studies [29], arranging capacitors around a common centroid helps minimize the impact of long-range gradients in oxide thickness. Additionally, dummy resistors are placed on both sides of the 7-bit RDAC to reduce resistor mismatch. This careful attention to layout details helps ensure the proposed ADC achieves high performance and accuracy.

## 4. Measurement Results and Discussion

**Figure 6:**
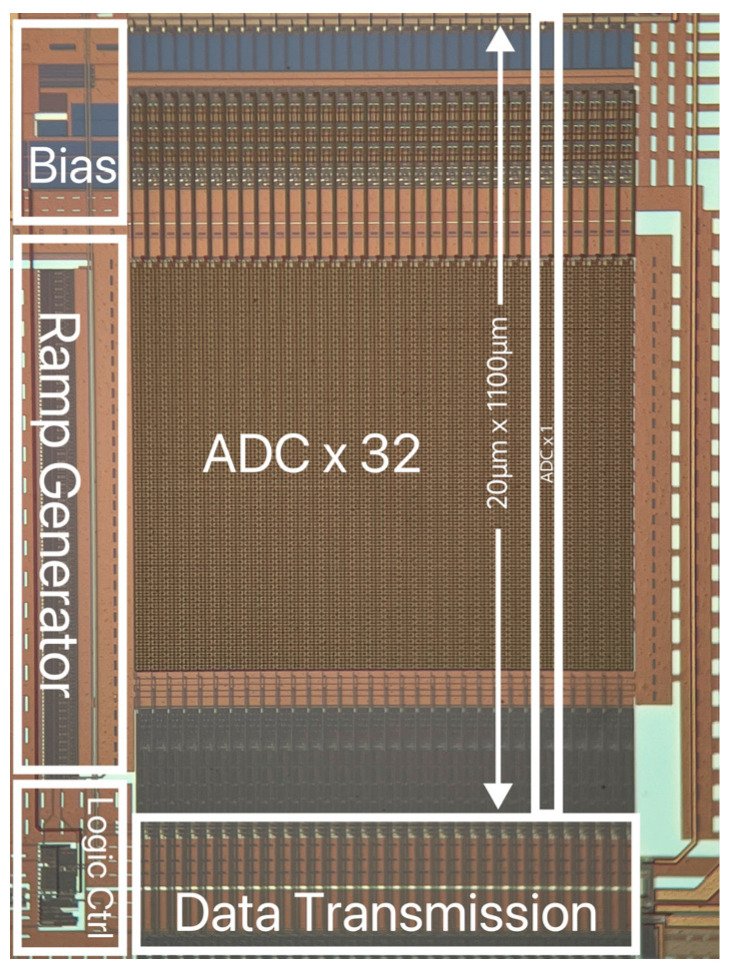
A microscope photo of the proposed ADC chip.

The proposed hybrid ADC arrays are fabricated in a 180 nm 1P6M CMOS process. The chip comprises 32 ADCs, an RDAC ramp generator, bias circuits, logic ctrl circuits, and data transmission circuits. The hybrid ADC occupies 20 µm × 1100 µm, as shown in Figure 6.

### 4.1. Measurement Setup

**Figure 7:**
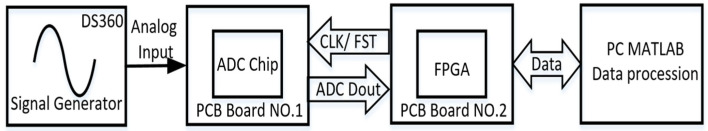
Block diagram of measurement setup.

**Figure 8:**
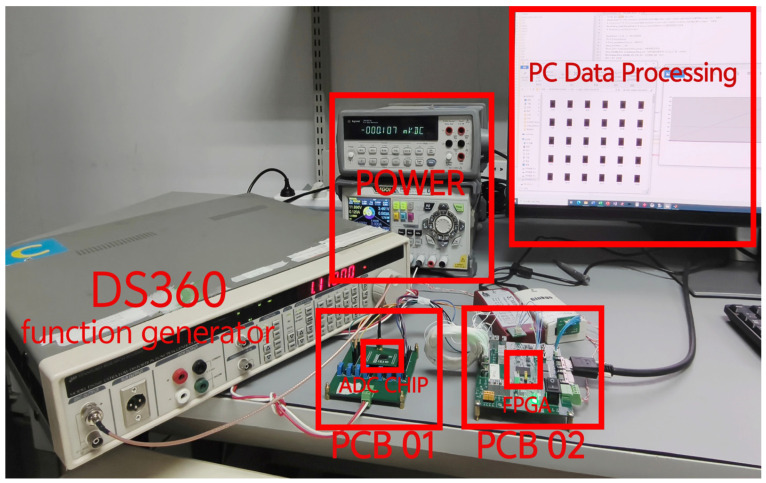
Measurement setup.

**Figure 9:**
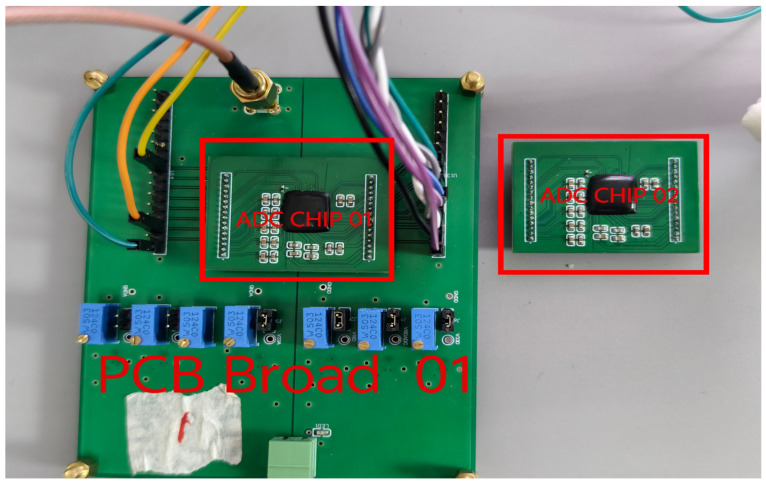
An image of the ADC chip and the custom-designed PCB for testing.

Figure 7 shows a block diagram of the measurement setup, and Figure 8 presents an image of the test measurement setup. Figure 9 illustrates the ADC chip mounted on the board along with our custom-designed PCB for ADC testing. The input signal from the DS360 waveform generator is fed to the PCB through an SMA connector. A customized power board labeled as No.1 provides the necessary power supply and reference voltages for both the analog and digital parts, and it drives the on-chip voltage buffers. An FPGA on board No.2 generates the clock and FST signals, and it captures the digital output data stream from the ADC output. These data streams are then exported to MATLAB for processing and computing the Fast Fourier Transform (FFT).

### 4.2. Measurement Results

**Figure 10:**
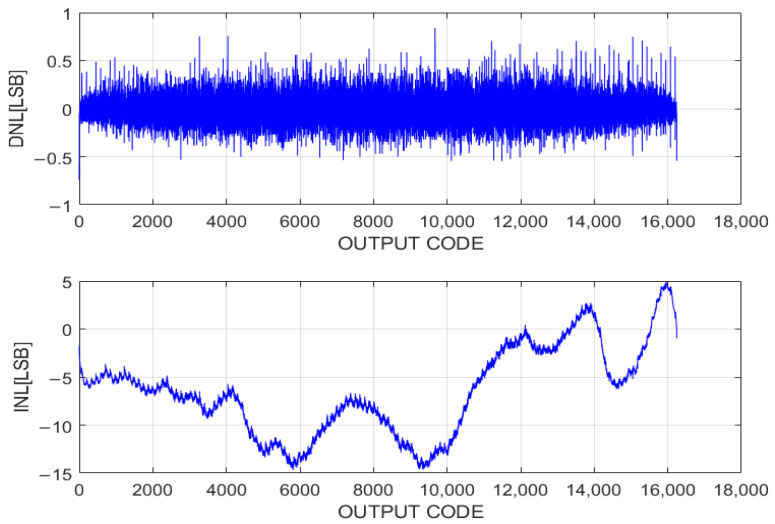
DNL and INL of proposed hybrid ADC.

At a 120 kS/s sampling rate, the measured DNL and INL of the proposed ADC are plotted in Figure 10. The measured DNL is −0.61 to +0.84 LSB without calibration, staying within ±1 LSB across the entire output range at a 14-bit resolution. In this design, a mismatch in the binary ratios of capacitors in the array and the kick-back noise from the comparator contribute to the DNL and INL errors. Since the single-ended method, as shown in Figure 1, the difference can be up to one-quarter of the supply voltage. It might cause a gain or INL error due to the comparator’s insufficient common-mode rejection ratio (CMRR) [30]. The peak-to-peak value of the INL is −14.5 to +4.8 LSB. However, for image sensor applications, INL errors have a negligible impact on photo quality [31,32,33].

**Figure 11:**
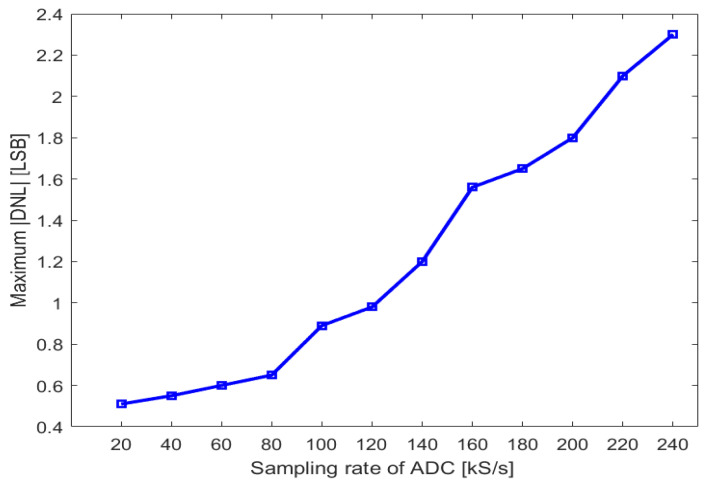
A plot of the maximum |DNL| as a function of the sampling rate.

In order to explore the influence of a higher sampling rate on the ADC, the DNL of the ADC at a higher sampling rate is tested. The curve in Figure 11 plots the maximum |DNL| as a function of the sampling rate. When the sampling frequency exceeds 120 kS/s, the maximum |DNL| value rises above 1. This increase in DNL errors is primarily due to the limited speed of the comparator. The proposed ADC achieves its best linearity and optimal FoM2 at the sampling rate of 120 kS/s.

**Figure 12:**
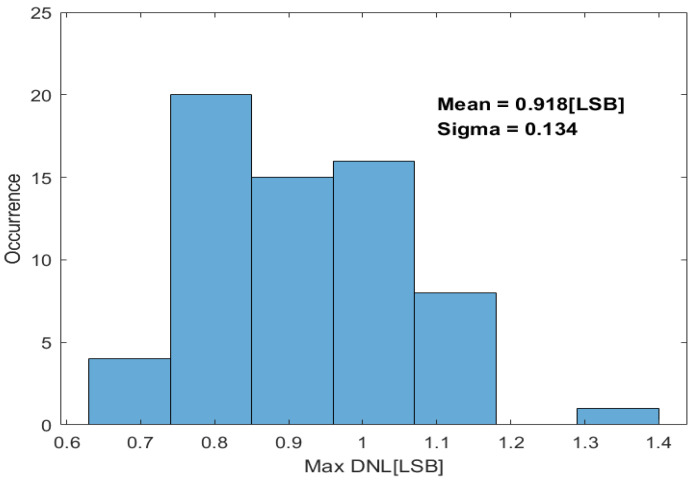
Measured maximum |DNL| of 64 ADC test samples.

A total of 64 ADC samples were tested, and the measurement results for the maximum |DNL| are presented in the histogram in Figure 12. The mean value of the maximum |DNL| is 0.918 LSB.

**Figure 13:**
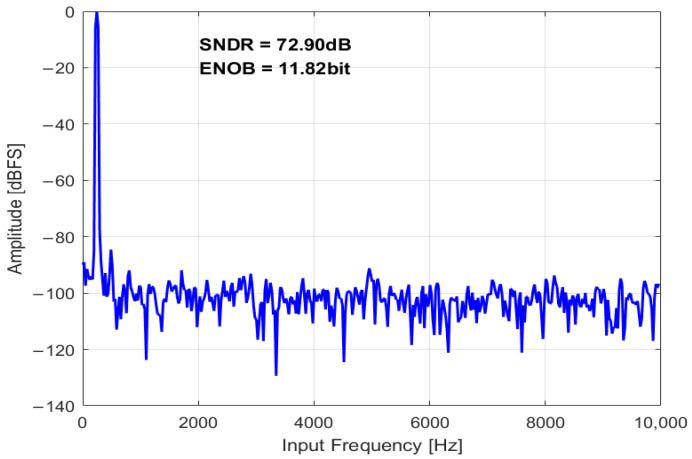
Measured FFT plot of proposed ADC.

To test the dynamic characteristics of the proposed ADC, a sinusoidal signal with a frequency of 344 Hz and V_peak-to-peak_ of 1.9 V is used as the input signal. Figure 13 shows the result of the 4096 points’ Fast Fourier Transformation (FFT). With a sampling rate of 120 kS/s, the ADC achieves a measured signal-to-noise and distortion ratio (SNDR) of 72.9 dB, corresponding to an effective number of bits (ENOB) of 11.82 bit. At a 14-bit resolution, 1 LSB corresponds to 115.9 μV with 1.9 V_FS_. At room temperature, the average value of the temporal noises is 2.5 LSB rms. At the liquid nitrogen temperature of 77 K for a cooled IRFPAS, the proposed ADC achieves a lower temporal noise of 1.7 LSB rms than at room temperature.

The analog circuits are powered by a 3.3 V supply, and the digital circuits are powered by a 1.8 V supply. At a sampling rate of 120 kS/s, the total power consumption of each proposed ADC is 71 μW. The power distribution is as follows: 40 μW for the comparator, 21 μW for the SAR logic and thermometer decoder, and 10 μW for the counter.

**Figure 14:**
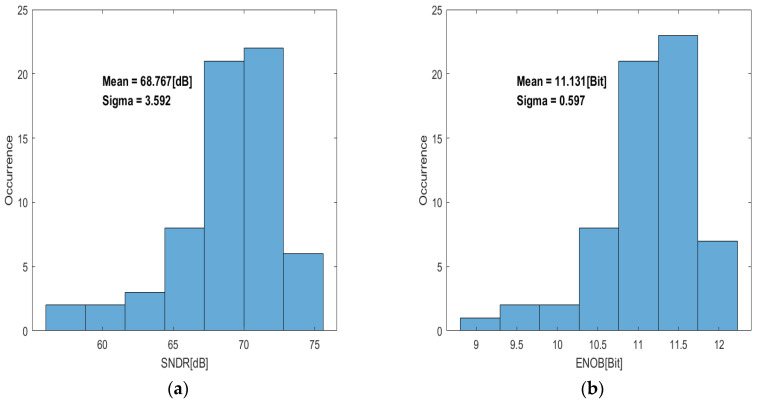
Measured dynamic performance of 64 ADC samples: (**a**) SNDR and (**b**) ENOB.

The histograms for the SNDR and ENOB of the 64 ADC samples are shown in Figure 14a,b, respectively. The mean SNDR is 68.76 dB, and the mean ENOB is 11.13 bits.

Table 1 summarizes the measured performance and compares it with earlier ADCs of similar architectures.

The figure of merits 1 (FoM1) is obtained from an equation in [21], and the FoM2 is obtained according to the Walden FoM equation [28]. These figures of merit are calculated to compare the power efficiency of the ADCs. Additionally, the Schreier figure of merit (FoMS) is calculated for a fair comparison across different resolutions. Since the comparator’s pre-amplifier uses a high supply voltage of 3.3 V, the power dissipation in this work is not the lowest. Consequently, it achieved a Walden FoM2 of 196.3 fJ/conv and a Schreier FoM_S_ of 162 dB. The proposed ADC also demonstrates high area efficiency with 6.08 μm^2^/code, making it suitable for small-pixel IRFPA applications. Furthermore, it achieved a good |DNL| < 1 without calibration at a sampling rate of 120 kS/s. However, due to mismatches and parasitic capacitance, the binary-weighted characteristics are not perfectly maintained. If the proposed ADC was to include comparator offset calibration techniques and error correction circuits, the ENOB and INL could be further improved, albeit at the cost of additional calibration circuitry.

## 5. Conclusions

This paper presents a 14-bit hybrid column-parallel ADC for small pixel pitch IRFPA applications. This hybrid ADC combines a 7-bit coarse SAR ADC with a 7-bit fine SS ADC, achieving a good balance between speed and power consumption. Only using a capacitor array for the 7-bit resolution to achieve a 14-bit ADC is a well-suited method for IRFPAs with small pixel pitches. Moreover, a tradeoff segmented thermometer-coded DAC is adopted in the first 7-bit coarse quantization, improving linearity and reducing the complexity of capacitor array matching.

A prototype chip with 32 ADCs was fabricated in a 0.18 μm process. A total of 64 ADC samples were tested. The ADC consumes 71 μW at a sampling rate of 120 kS/s, achieving an FoM_W_ of 196.3 fJ/conv and an FoM_S_ of 162 dB. Thanks to the segmented thermometer DAC, the proposed hybrid ADC achieves a differential nonlinearity of −0.48/+0.94 LSB without calibration or redundancy bits. The proposed ADC is suitable for small pixel pitch IRFPA readout applications. It has a high resolution and is power-efficient and area-efficient, making it suitable for digital readout integrated circuits for SWaP-constrained applications.

## Figures and Tables

**Table 1:** Performance comparison with previous works.

	[21] TED 2016	[22] VLSI 2022	[20] Sensor J 2020	[23] ISCAS 2020	[34] Electron 2022	This Work
Process	90 nm	130 nm	180 nm	180 nm	28 nm	180 nm
Supply voltage (V)	2.8/1.2	3.3/1.2	2.8/1.5	1.8	N/A	3.3/1.8
Architecture	SAR/SS	TS SS	TS SS	SAR/SS	SAR/SS	SAR/SS
Resolution (bit)	12	12	12	10	14	14
ENOB (bit)	N/A	9.8	9.13	9.1	10.81	11.82
Conversion time (μs)	2.7	10	39.7	0.8	2.8	8.3
Power (μW)	56	62	6.53	36	34	71
DNL (LSB)	−0.45/+0.84	−1/+0.83	−1/+4.25	−0.61/+0.6	−1/+2.5	−0.61/+0.84
INL (LSB)	−1.5/+0.74	−3.31/+4.78	−7.3/+5.73	−0.89/+0.82	−3.3/+3.6	−14.5/+4.8
* FoM1 (fJ/conv)	36.9	151.3	1.75	87.89	5.8	43.3
** FoM2 (fJ/conv)	N/A	695.5	447	52.5	53	196.3
*** FoM_S_ (dB)	N/A	149.6	149.4	158.8	163.8	162
Area(μm^2^)	2.24 × 998	7.5 × 675	5.6 × 1007	60 × 200	550 × 1180	20 × 1100
**** AE(μm^2^/code)	N/A	5.68	10.6	21.8	361	6.08

* FoM1 = [ Power]/([1/f_s_] × 2^[Resolution]). ** FoM2 = [Power]/([1/f_s_] × 2^[ENOB]). *** FoM_S_ = SNDR (dB) + 10log(Bandwidth/[Power]) = (6ENOB + 1.8) (dB) + 10log(Fs/2/[power]). **** AE = [ Area]/(2^[ENOB]).

## Data Availability

Data are contained within the article.
